# Supplementary figures and images for: Variants in GSTZ1 Gene Underlying Maleylacetoacetate Isomerase Deficiency: Characterization of Two New Individuals and Literature Review
Source: Genes (Basel). 2025 Aug 26;16(9):1009. doi: 10.3390/genes16091009 (PMC12470001; doi:10.3390/genes16091009)

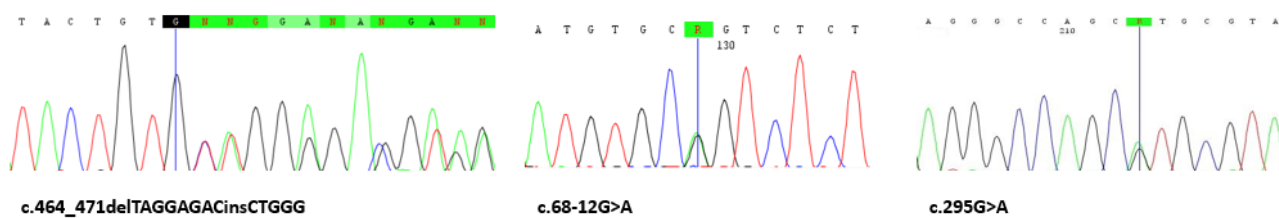

Supplement: Supplementary file 1 [file genes-16-01009-s001.zip › Figure S2.pdf]

A

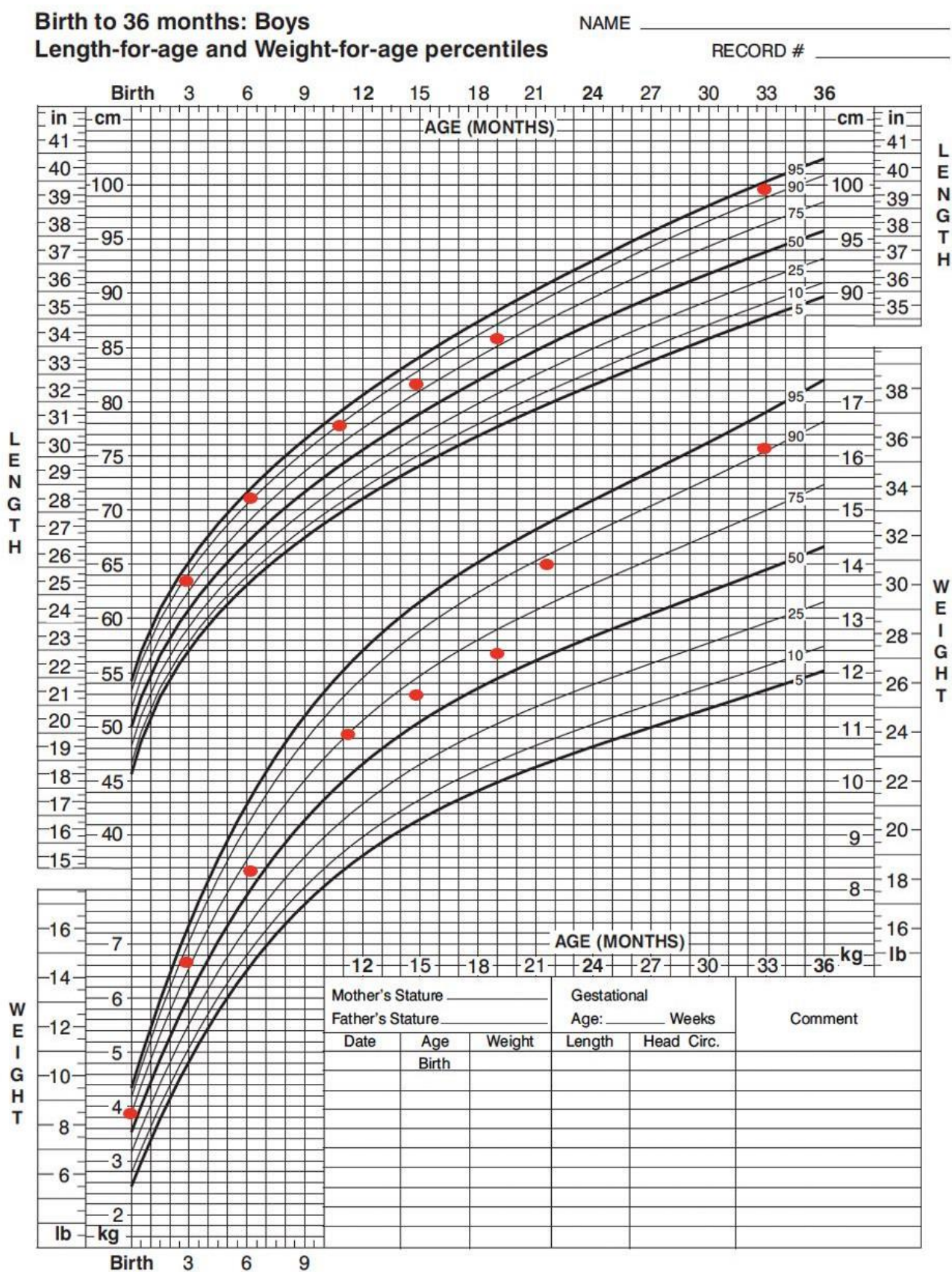

# 2 to 20 years: Boys

## Stature-for-age and Weight-for-age percentiles

NAME \_\_\_\_\_

RECORD # \_\_\_\_\_

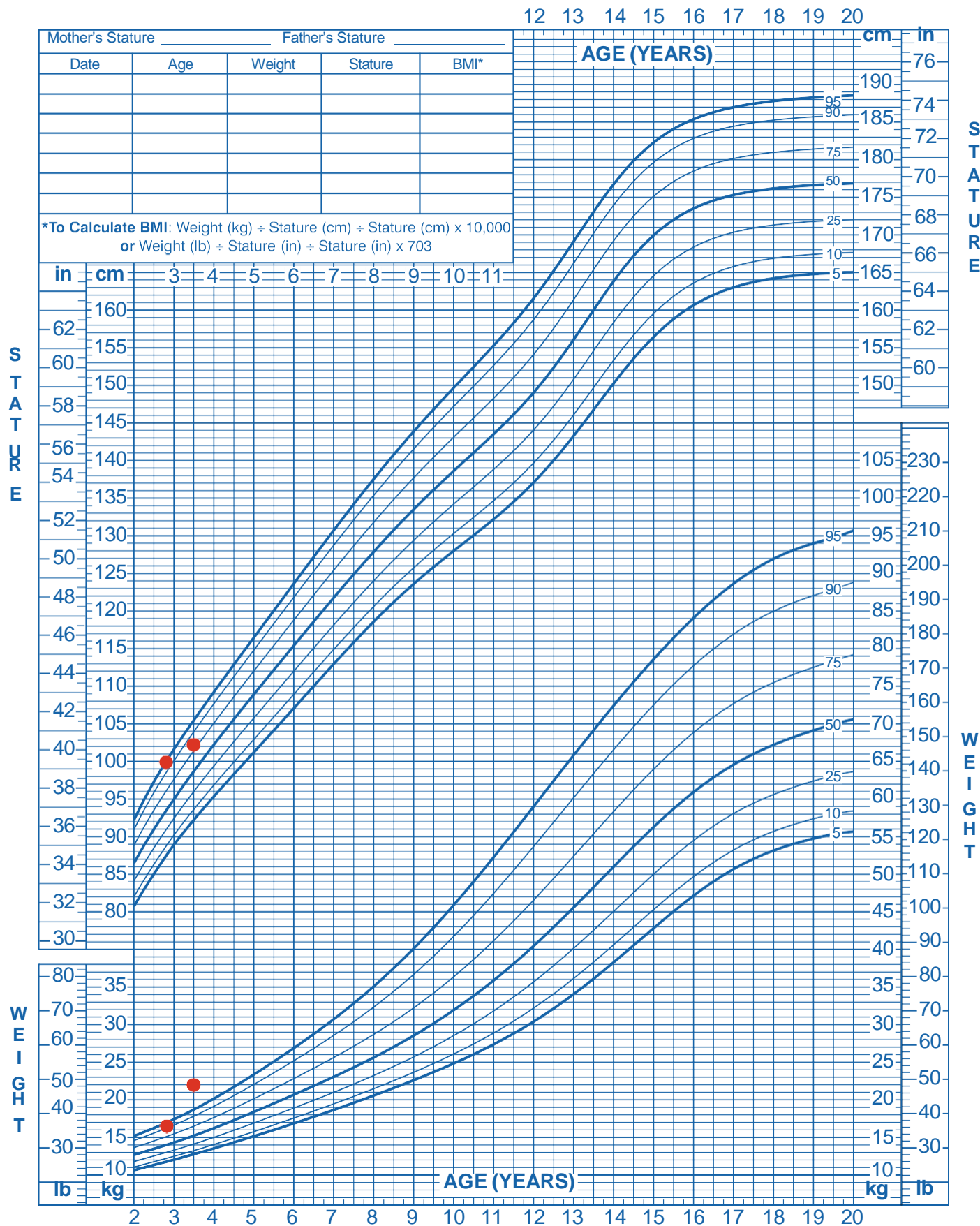

Supplement: Supplementary file 1 [file genes-16-01009-s001.zip › Figure S1.pdf]
